# Effects of Interface Oxidation on Noise Properties and Performance in III–V Vertical Nanowire Memristors

**DOI:** 10.1021/acsami.2c21669

**Published:** 2023-04-07

**Authors:** Mamidala Saketh Ram, Johannes Svensson, Lars-Erik Wernersson

**Affiliations:** Department of Electrical and Information Technology, Lund University, 221 00 Lund, Sweden

**Keywords:** nanowire, memristors, RRAMs, 1T1R, III−V, low-frequency noise, InAs

## Abstract

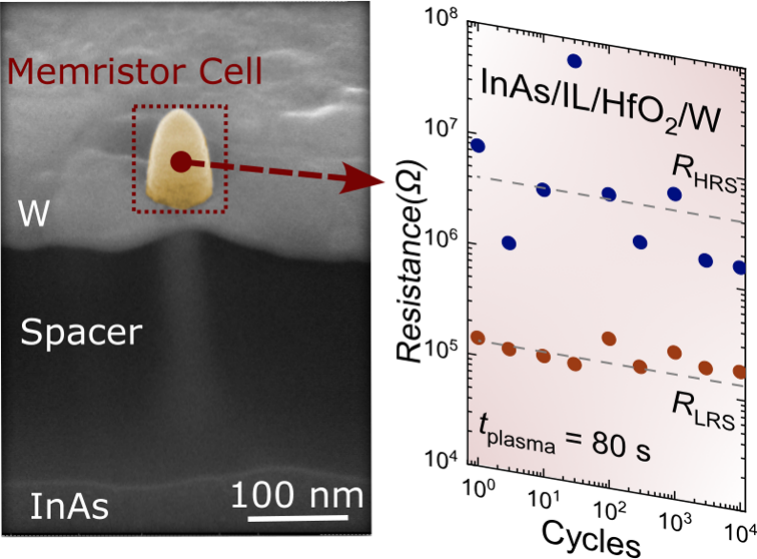

Memristors implemented as resistive random-access memories (RRAMs) owing to their low power consumption, scalability, and speed are promising candidates for in-memory computing and neuromorphic applications. Moreover, a vertical 3D implementation of RRAMs enables high-density crossbar arrays at a minimal footprint. Co-integrated III–V vertical gate-all-around MOSFET selectors in a one-transistor-one-resistor (1T1R) configuration have recently been demonstrated where an interlayer (IL)-oxide has been shown to enable high RRAM endurance needed for applications like machine learning. In this work, we evaluate the role of the IL-oxide directly on InAs vertical nanowires using low-frequency noise characterization. We show that the low-frequency noise or the 1/*f*-noise in InAs vertical RRAMs can be reduced by more than 3 orders of magnitude by engineering the InAs/high-*k* interface. We also report that the noise properties of the vertical 1T1R do not degrade significantly after RRAM integration making them attractive to be used in emerging electronic circuits.

## Introduction

Deep neural networks (DNNs) in recent times have shown great promise in machine learning applications such as image classification and object detection for autonomous driving.^1,2^ The need for increased DNN accuracy leads to an increased demand for scaled, faster, and more energy-efficient hardware.^2^ However, the traditional hardware is based on the von Neumann architecture where the memory and computing units are separate, thus limiting the performance dictated by the rate of data exchange.^3^

A promising solution to overcome the von Neumann bottleneck is in-memory computing where memristor cells such as resistive-random-access-memories (RRAMs) are integrated into dense crossbar arrays.^4^ The weights used for instance in DNNs can be stored in RRAM elements and computation can be performed using multiply-and-accumulate operations.^2^ The RRAM elements integrated into a one-transistor-one-resistor (1T1R) configuration can enable crossbar arrays in a cost and energy-efficient way. The 1T1R configuration has recently been utilized in power-efficient signal processing using reservoir computing and has also been used to demonstrate Boolean logic capabilities beneficial for in-memory computing.^3−6^

Using a metal-oxide-semiconductor-field-effect-transistor (MOSFET) selector in crossbar arrays would reduce current sneak paths and provide a compliance current level controlled by the MOSFET gate. A robust compliance current is crucial in preventing current overshooting or a hard breakdown of the RRAM cell.^7^ The adjustable compliance current level offered by a MOSFET selector also enables multi-bit operation of the RRAM cell further increasing the memory density.^8^ However, state-of-the-art FinFETs, due to their planar geometry and relatively large footprint, are not suited to be densely integrated into crossbar arrays.

To overcome the concern of a relatively large footprint, vertical gate-all-around (GAA) MOSFETs using Si, and recently III–V materials, due to favorable transport properties and voltage scalability, have been demonstrated as suitable selector devices.^3,7,9,10^ The vertical 1T1R geometry allows for a footprint of 4*F*^2^, where *F* is the minimal distance between two metal lines in a crossbar array.

In our previous study, we demonstrated a fully vertical 1T1R with an RRAM endurance of 10^6^ cycles capable of performing Boolean logic operations with a theoretical minimal footprint of 4*F*^2^.^3^ The reported vertical 1T1R comprises an InAs/interlayer (IL)-oxide/HfO_2_/W RRAM and an InAs/Al_2_O_3_/HfO_2_/TiN+W MOSFET gate stack. It was noted in our previous study that the IL-oxide thickness and composition are crucial design parameters to enable high RRAM endurance. The IL-oxide properties were tuned by controlled oxidation of the vertical nanowire (VNW) InAs interface. The thickness and composition of the IL-oxide were determined using XPS performed at the FlexPES beamline of MAX IV in Lund, Sweden, on large planar samples as characterization of extremely small cell area dimensions (*A*_cell_ ∼ 0.01 μm^2^) on nanowires using XPS is challenging.^3^ Although the thickness and composition of the IL-oxide were determined, the role of the IL-oxide in RRAM switching was not fully understood.

In this work, we provide a deeper understanding of the IL-oxide using low-frequency noise (LFN) and random telegraph noise (RTN) characterization. Additionally, the LFN characterization technique allows for the evaluation of the IL-oxide present in highly scaled RRAM cells directly on VNWs.

It is known that noise in circuits implemented using non-volatile memory crossbars can accumulate causing large output errors.^4^ Notably, the 1/*f*-noise, which is a dominant source of noise in electronic circuits, can be reduced by over 3 orders of magnitude by controllable oxidation of the InAs/high-*k* interface. We also find that the vertical GAA MOSFET selector integration does not degrade the noise properties of the 1T1R cell significantly making them suitable to be used in in-memory computing circuits.

## Experimental Section

**Figure 1:**
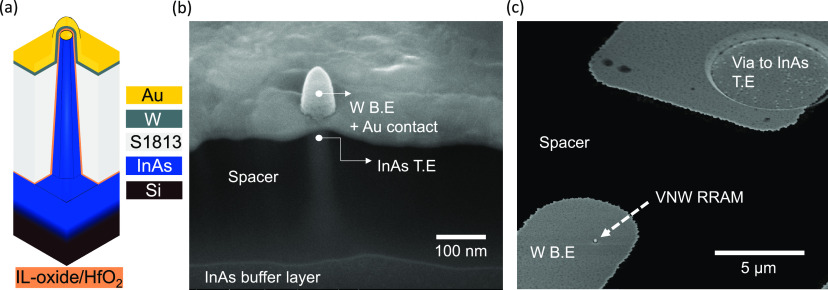
(a) Cross-sectional schematic of the VNW InAs/IL-oxide/HfO_2_/W RRAM where the NW height after growth is ∼450 nm and the diameter is ∼40 nm. (b) Cross-sectional and (c) top-view scanning electron microscope image of the fabricated VNW InAs/ IL-oxide HfO_2_/W RRAM indicating the RRAM top electrode (T.E.) and bottom electrode (B.E).

A cross-sectional illustration and a scanning electron microscope (SEM) image of the fabricated VNW InAs/IL-oxide/HfO_2_/W RRAM is shown in Figure1. P-type Si (111) was used as the starting substrate on which a 300 nm-thick InAs buffer layer was grown using metal–organic vapor-phase epitaxy. VNWs were grown using the vapor–liquid–solid growth method with the help of Au seed particles patterned by electron-beam lithography. The InAs VNW acts as the RRAM top electrode allowing for a 3D 1T1R configuration. For the RRAM switching dielectric, 2.8 nm-thick HfO_2_ was deposited using plasma-enhanced atomic layer deposition (PEALD) at 200 °C. An IL oxide was formed by using an oxygen plasma length (*t*_plasma_) of 80 s for the first 10 PEALD cycles. To study the properties of the IL-oxide on the VNW, samples with three different oxygen plasma lengths, *t*_plasma_ = 10, 30, and 80 s, with the corresponding IL-oxide thickness estimated to be ∼1.5, 2, and 3 nm were fabricated. To define the RRAM cell area, a resist spacer (S1813) is first spin-coated and then thinned down using an oxygen plasma dry etch until the top segment (100 nm) of the InAs nanowire is exposed. A 20 nm-thick W RRAM bottom electrode followed by 100 nm-thick Au for contacts is sputtered and patterned using photolithography. The process flow illustrating the key process steps is shown in Figure S1.

## Results and Discussion

### Direct Current (DC) and Pulsed Characterization

**Figure 2:**
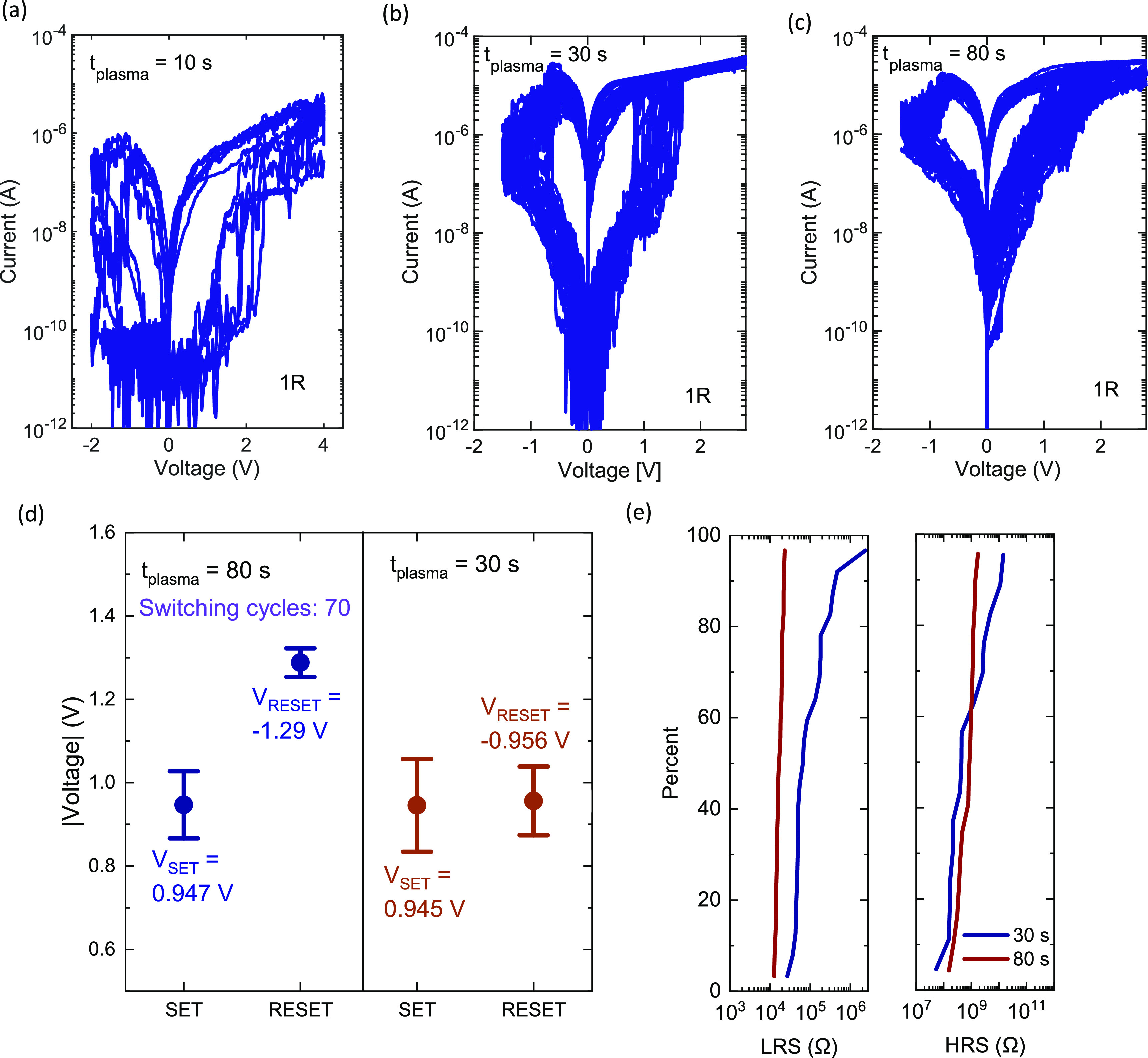
(a) *I*–*V* characteristics of the InAs VNW RRAM for *t*_plasma_ = 10 s. (b) *I*–*V* characteristics of the InAs VNW RRAM for *t*_plasma_ = 30 s. (c) *I*–*V* characteristics of the InAs VNW RRAM for *t*_plasma_ = 80 s. (d) Switching voltage statistics for both plasma length samples (*t*_plasma_ = 30 s and *t*_plasma_ = 80 s). (e) Cumulative distribution plot of resistance states for both plasma length samples (*t*_plasma_ = 30 s and *t*_plasma_ = 80 s).

DC characterization of InAs/IL-oxide/HfO_2_/W RRAM stack on InAs VNWs in a 1R configuration before the 1T1R integration was performed using an Agilent B1500A parameter analyzer. The initial filament forming voltage (*V*_FORM_) as shown in Figure S2 was measured to be ∼3.0 V. Figure 2a–c shows the switching *I*–*V* characteristics for the InAs VNW RRAM with plasma lengths, *t*_plasma_ = 10 s, *t*_plasma_ = 30 s, and the longest plasma length, *t*_plasma_ = 80 s. From the *I*–*V* characteristics, it can be noted that for the shortest plasma length sample, the RRAM switching has a larger cycle-to-cycle variation when compared to the longest plasma length sample. To evaluate the DC switching variability as a function of plasma length, RRAM statistics for switching voltages and resistance states were gathered for an intermediate plasma length, *t*_plasma_ = 30 s, and compared to *t*_plasma_ = 80 s. The switching voltages (SET and RESET) for 70 DC switching cycles are shown in Figure 2c, where the plotted point is the median value and the bars represent a 95% confidence interval. Figure 2d shows the cumulative distribution plots for the low-resistive state (LRS) and the high-resistive state (HRS) with either plasma length. The LRS and HRS were determined using a read voltage (*V*_READ_) of −50 mV, a stop voltage (*V*_STOP_) of −1.5 V < *V*_STOP_ < 3.0 V, and a compliance current of 30 μA. From both figures, it can be noted that the spread in switching voltages and resistance states is lowered with increasing *t*_plasma_. The improvement in switching stability with increasing *t*_plasma_ is attributed to a reduction of trap sites near the InAs interface. It has been previously observed that the activation energy (*E*_A_) for trap sites in RRAMs varies cycle-to-cycle as well as device-to-device affecting device reliability.^11^ The spread in InAs VNW RRAM switching voltages and resistance states can further be improved by integrating a transistor selector in a 1T1R configuration.^3^ A detailed explanation of the cause of the spread in switching data is described in the next section.

**Figure 3:**
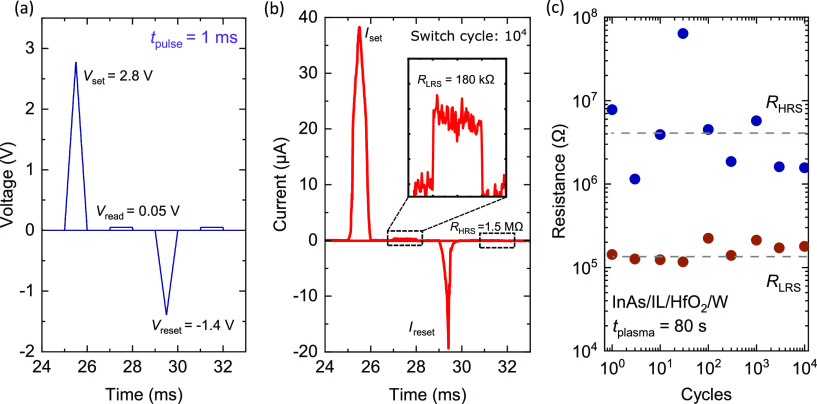
(a) Voltage pulse-train applied to the VNW RRAM. (b) Measured current after switching the VNW RRAM for 10^4^ cycles. (c) Endurance measurement demonstrating both resistance states for 10^4^ cycles.

To also evaluate reliability before selector integration, pulsed endurance measurements on the VNW RRAM with the longest *t*_plasma_ were performed using an Agilent B1500A waveform generator/fast measurement unit. The applied voltage pulse train consisting of a SET-READ-RESET-READ pulse sequence is shown in Figure 3a. A relatively long pulse width (*t*_pulse_) of 1 ms was used for initial performance validation. Triangular voltage pulses were used for SET/RESET to minimize current overshoots due to parasitic capacitances.^12^ The measured current for the applied voltage pulse train is shown in Figure 3b where it can be noted that *I*_SET_ and *I*_RESET_ are below 40 μA demonstrating scaled CMOS selector compatibility.^8^ The switching resistances were measured by applying 50 mV READ pulses. Notably, the InAs VNW RRAM after interface oxidation (*t*_plasma_ = 80 s) demonstrated an endurance of 10^4^ switching cycles as shown in Figure 3c. The larger spread in the HRS (*R*_HRS_), when compared to the LRS (*R*_LRS_) is attributed to not having a current limiting selector device in series. This leads to an uncontrolled parasitic filament growth in the switching oxide due to current overshooting as shown in Figure S3. However, the current overshoot can be mitigated using an integrated selector device, which can effectively limit the current delivered to the RRAM.^7^ For the InAs/IL-oxide/HfO_2_/W RRAM, we have previously demonstrated a reduction in the spread in *R*_HRS_ and an increase in endurance from 10^4^ cycles to 10^6^ cycles by integrating a current limiting MOSFET selector.^3^ An endurance of at least 10^5^ cycles is sufficient for basic machine-learning applications.^13^

Although it is possible for the RRAM performance to further improve for *t*_plasma_ > 80 s, we have not studied the effect of plasma time exposure on the IL-oxide growth saturation in this work.

### Low-Frequency Noise Analysis

It is well-known in MOSFETs that the trapping/de-trapping of electrons from the channel to the interface traps in the gate dielectric results in 1/*f* noise in the drain current, which is explained using the McWhorter model.^14^ Inspired by the Mcwhorter model, LFN or 1/*f* noise in RRAMs is shown to be a result of electron tunneling between the electrode and the traps in the dielectric.^15^

**Figure 4:**
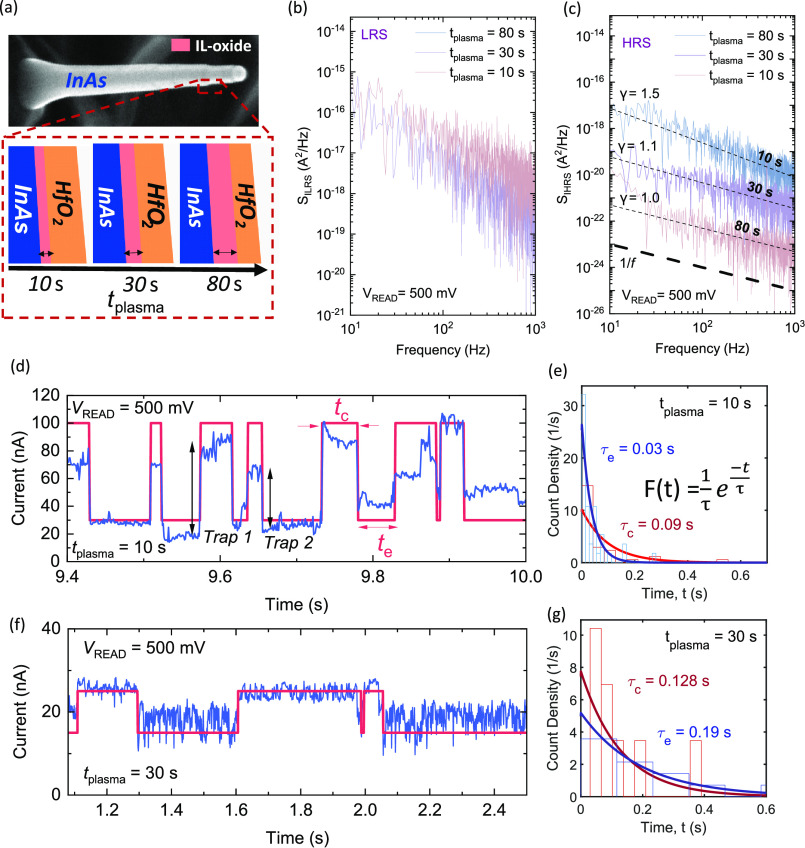
(a) SEM image of a vertical InAs nanowire, and an illustration of different stages of IL-oxide growth. (b) Current noise spectral density of the vertical nanowire RRAM in the LRS. (c) Current noise spectral density of the vertical nanowire RRAM in the HRS. (d) RTN characterization for *t*_plasma_ = 10 s. (e) Histogram for capture and emission times for *t*_plasma_ = 10 s. (f) RTN characterization for *t*_plasma_ = 30 s. (g) Histogram for capture and emission times for *t*_plasma_ = 30 s.

LFN measurements at room temperature were carried out to evaluate the IL-oxide present at the InAs/HfO_2_ interface directly on the InAs VNW as shown in Figure 4a. Table 1 summarizes the XPS findings on planar structures, and it can be noted that the IL-oxide primarily consists of In_2_O_3_ and As_2_O_3_, which initially is As_2_O_3_ rich at the InAs/HfO_2_ interface for the shortest plasma length, *t*_plasma_ = 10 s. In contrast, a more In_2_O_3_-rich, balanced IL is obtained with increasing *t*_plasma_.^3^

**Table 1:** XPS Analysis of IL-Oxide on Planar Samples

*t*_plasma_ (s)	IL-oxide thickness (Å)	IL composition
10	∼15	As_2_O_3_ rich
30	∼20	increasing In_2_O_3_
80	∼30	balanced In_2_O_3_ and As_2_O_3_

Noise measurements were carried out using a semiconductor parameter analyzer (B1500A) using a wave generator fast measurement unit module. A fast current measurement was performed where the RRAM current (*I*_RRAM_) was evaluated over 10 s using RSUs (remote sense/switch units). A schematic of the setup used is shown in Figure S4. The InAs VNW RRAM top electrode (InAs) was biased at a constant read voltage, *V*_READ_ = 500 mV, while the bottom electrode (W) was grounded. The current noise power spectral density (*S*_I_) was estimated using fast Fourier transform by employing the Welch method.^16^ The LFN measurement was performed for both the LRS and the HRS. When the RRAM is in the LRS, it can be observed that although the current noise power spectral density, *S*_ILRS_ follows a 1/*f* dependency as shown in Figure 4b, there is no significant difference in *S*_ILRS_ between devices with varying IL-oxide thicknesses. This is due to ohmic conduction through the oxygen vacancy filament dominating at higher current levels (μA range). Notably, when the RRAM is switched to its high-resistive state, the current noise power spectral density, *S*_IHRS_ reduces distinctly with increasing IL-oxide thickness as shown in Figure 4c. Besides the decrease in noise, it can also be observed in the HRS that while having 1/*f*^γ^ dependency, where γ = −∂ln*S*_I_/∂ln*f*, the value of γ changes from 1.5 to 1.0. A gamma value typically in the range of ∼1 indicates a distribution of traps in the oxide, which is uniform in depth and does not have any dominant contribution.^17−20^ A higher gamma value might be understood as an increase in trap density due to the formation of a thin highly defective IL-oxide as shown in Figure 4a. As a reference, a controllable reduction in oxygen defect density by introducing more oxygen is also observed in semiconducting oxide-based transistors, where a high density of mobile oxygen defects (vacancies) degrade the transistor performance.^21^

The metal/semiconductor interface is widely explained using the charge neutrality level.^22^ Similarly, the III–V/dielectric interface is described using the so-called trap neutral level (TNL).^23^ For an In_2_O_3_ film thicker than 1.5 nm, the TNL is known to lie 0.4 eV above the conduction band (*E*_c_) making it a conductive oxide.^24^ Similarly, the TNL is shown to be 0.2 eV below the conduction band when 1.5 nm thick and continuously moves down into the bandgap with decreasing thickness. It can be understood that as the IL-oxide grows thicker than 1.5 nm (*t*_plasma_ > 10 s) and turns more conductive, it may interact with the InAs to form the switching electrode. On the other hand, when the IL-oxide thickness is less than 1.5 nm (*t*_plasma_ = 10 s), the insulating IL is very thin (atomic layers) and highly defective making a part of the HfO_2_ switching dielectric. The tuning of the IL-oxide *E*_F_ above the *E*_c_ for *t*_plasma_ = 80 s also increases the vacancy formation energy preventing cycle-to-cycle vacancy migration during RRAM switching.^25^

To evaluate the RRAM top electrode Fermi energy level position and its modulation with respect to the defect energy level in the switching dielectric, we measured random-telegraph-noise (RTN) characteristics. The RTN measurement was performed for RRAMs with *t*_plasma_ = 10 s, *t*_plasma_ = 30 s, and *t*_plasma_ = 80 s in the HRS using *V*_READ_ = 500 mV. RTN is observed as distinct changes in the measured current over time due to electron trapping/de-trapping events. Notably in Figure S5, no RTN was observed for *t*_plasma_ = 80 s at *V*_READ_ = 500 mV due to a likely reduction in trap density with increased oxidation. Figure 4d,f shows the trapping and de-trapping events for HRS currents versus time for samples with *t*_plasma_ = 10 s and *t*_plasma_ = 30 s. The characteristic capture time constant (τ_c_) and emission time constant (τ_e_) as shown in Figure 4e,g were determined by fitting an exponential distribution of the capture time (*t*_c_) and emission time (*t*_e_) indicated in Figure 4d.^26^ The capture time constant and emission time constant for IL-oxide with *t*_plasma_ = 10 s and *t*_plasma_ = 30 s were determined to be τ_c_ = 0.09 s, τ_e_ = 0.03 s and τ_c_ = 0.128 s, τ_e_ = 0.19 s respectively. When *t*_plasma_ = 10 s, we observe that τ_*c*_ > τ_*e*_, and when *t*_plasma_ = 30 s, we find τ_e_ > τ_c_ indicating the upward movement of the Fermi-level of IL-oxide with respect to the defect energy level.^27^

It can be observed from Figure 4d that the RTN is caused by more than a single trap indicating a higher trap density when *t*_plasma_ = 10 s. The participation of at least two traps is indicated in Figure 4d, where the change in current (Δ*I*) after an electron is trapped for trap 1 and trap 2 is different, whereas when an individual defect is dominant for causing RTN, Δ*I* after charge trapping remains the same.^26^ The presence of more than one trap makes it difficult to determine exactly a characteristic trap time constant but τ_*c*_ > τ_*e*_ changing to τ_e_ > τ_c_ with increasing IL-oxide thickness nevertheless indicates increased conductivity of the IL-oxide.

The increased LFN along with a modulation of the IL-oxide Fermi-level into the bandgap for *t*_plasma_ = 10 s most likely explains the observed larger spread in RRAM switching voltages and resistance states, which are not desirable for high endurance operation. More importantly, the LFN characterization technique can be utilized as an easy tool to evaluate and tune the interface properties for extremely scaled RRAM cells directly on a VNW.

Finally, LFN measurements were also performed on a fabricated InAs VNW 1T1R cell with an RRAM cell area of 0.01 μm^2^ and selector gate length (*L*_G_) of 200 nm to evaluate the influence of RRAM integration on the vertical GAA MOSFET selector LFN properties. To fabricate the 1T1R structure, the selector gate stack consisting of an Al_2_O_3_/HfO_2_ bilayer dielectric is integrated on the same InAs VNW containing the RRAM stack.^3^ Although in the demonstrated 1T1R structure, HfO_2_ is used as the switching dielectric for the RRAM and gate dielectric for the MOSFET selector, the deposition process used for both is different. PEALD is used to deposit the RRAM HfO_2_, where oxygen plasma pulses are used to oxidize the InAs interface. On the other hand, as native oxides are detrimental to MOSFET performance, thermal atomic layer deposition (TALD) of HfO_2_ is used during which the InAs surface is passivated using TMA precursor pre-dosing. It is well-known that the self-cleaning effect during TALD and TMA surface passivation removes native oxides and improves the InAs/HfO_2_ interface.^28−30^

**Figure 5:**
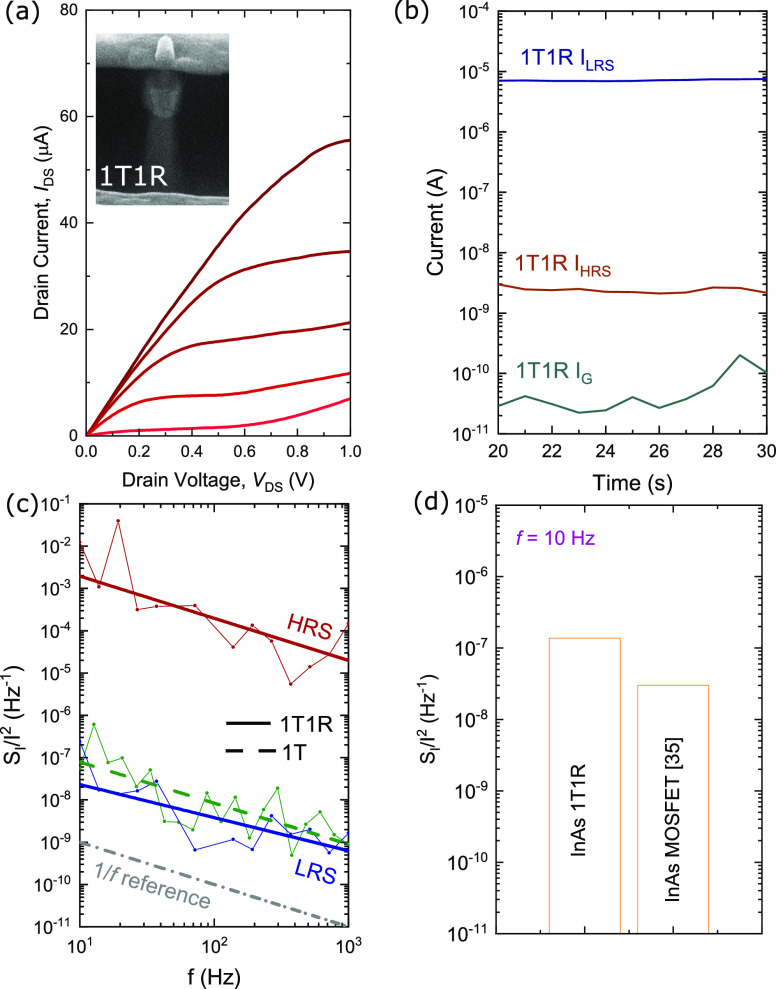
(a) Cross-sectional SEM image of a InAs VNW GAA 1T1R and measured selector output characteristics (−1.0 V < *V*_GS_ < 1.0 V, Δ*V*_GS_ = 0.5 V). (b) Measured current for both RRAM resistance states (LRS and HRS) and MOSFET selector gate leakage. (c) Current noise spectral density for the 1T1R with the integrated RRAM and for a standalone vertical MOSFET in the ON-state without RRAM integration. (d) Comparison of the current noise spectral density at *f* = 10 Hz for the 1T1R and previously reported InAs MOSFET.

A cross-sectional SEM image of the 1T1R cell and the integrated GAA MOSFET selector output characteristics are shown in Figure 5a. It can be noted that there is a non-ideal increase in the drain-to-source current (*I*_DS_) at *V*_DS_ = ∼0.6 V. The increase in current is due to the onset of impact ionization and band-to-band tunneling. The increase in current was also observed in previous experimental demonstrations of VNW III–V MOSFETs, where a breakdown typically occurs at *V*_DS_ = 0.7 V.^18,31,32^ The onset of increasing current can be extended beyond 1.0 V by fabricating a vertical field-plate on the drain side.^31^

It can be noted from Figure 5a that the GAA MOSFET selector gate after RRAM integration can efficiently modulate the current delivered to the RRAM cell placed on top of the nanowire. Figure 5b shows the current levels for the integrated RRAM LRS (*I*_LRS_), HRS (*I*_HRS_), and the selector gate current (*I*_G_). An *I*_G_ of 20 pA indicates negligible selector gate leakage current and an *I*_HRS_/*I*_LRS_ > 1000 is a sufficiently large RRAM memory window. The *I*_LRS_, *I*_HRS_, and *I*_G_ were measured with *V*_GS_ = 1.0 V and *V*_READ_ = 0.1 V.

For LFN measurements on the 1T1R cell, the current noise power spectral density was recorded using a lock-in amplifier while supplying a constant *V*_READ_ of 100 mV using a low-noise preamplifier. This setup as shown in Figure S6 was particularly used to measure the 1T1R cell in a similar way as done in previous LFN measurements on VNW III–V MOSFETs.^18,33^ This would allow for a direct comparison of current noise power spectral density (*S*_I_) between the III–V VNW 1T1R cell and the 1T III–V VNW MOSFET to evaluate the impact of RRAM integration on noise properties.

Figure 5c shows the current noise power spectral density of the measured current for the VNW 1T1R and is compared to the standalone VNW 1T without RRAM integration. Notably, when the integrated RRAM is in the LRS and the applied voltage falls across the MOSFET selector, the noise power spectral density is measured to be similar to that of the standalone VNW 1T when biased in the ON-state. The current noise power spectral density at 10 Hz was measured to be 1.3 × 10^–7^ Hz^–1^ and is also not significantly different when compared to previously reported *S*_I_ of vertical InAs nanowire MOSFET (1T) as shown in Figure 5d.

As LFN is a dominant source of noise in modern electronic circuits,^34,35^ our measurements indicate that the RRAM integration does not result in significant degradation of LFN properties of the vertical 1T1R cell, making it attractive for circuit applications.

## Conclusions

In this work, we present LFN characterization as a tool to evaluate the IL-oxide directly on VNWs, which is needed for high-endurance resistive switching. We report that the LFN or the 1/*f*-noise in vertical RRAMs can be reduced by more than 3 orders of magnitude by engineering the nanowire/high-*k* interface. We also find that vertical GAA MOSFET selector integration does not result in significant degradation of LFN properties of the 1T1R structure making it suitable for in-memory computing applications.

## Supporting Information Available

Fabrication flow diagram of the VNW RRAM, a biasing scheme for forming the VNW RRAM, measured current to show the overshooting problem, measurement setup using RSUs for low-frequency noise characterization, RTN measurement for the sample with longest interface oxidation, lock-in amplifier setup, and noise measurement comparison of the lock-in amplifier and RSU units (PDF)
