# Supplementary material for: Effects of Interface Oxidation on Noise Properties and Performance in III–V Vertical Nanowire Memristors
Source: ACS Appl Mater Interfaces. 2023 Apr 7;15(15):19085–91. doi: 10.1021/acsami.2c21669 (PMC10119853; doi:10.1021/acsami.2c21669)
Supplement: Supplementary file 1 — am2c21669_si_001.pdf [file am2c21669_si_001.pdf]

Supporting Information:

Effects of Interface Oxidation on Noise Properties and Performance in III-V Vertical Nanowire Memristors

Mamidala Saketh Ram*‡, Johannes Svensson‡, and Lars-Erik Wernersson‡

‡Electrical and Information technology, Lund University, Box 118, Lund 221 00, Sweden

Email: saketh_ram.mamidala@eit.lth.se

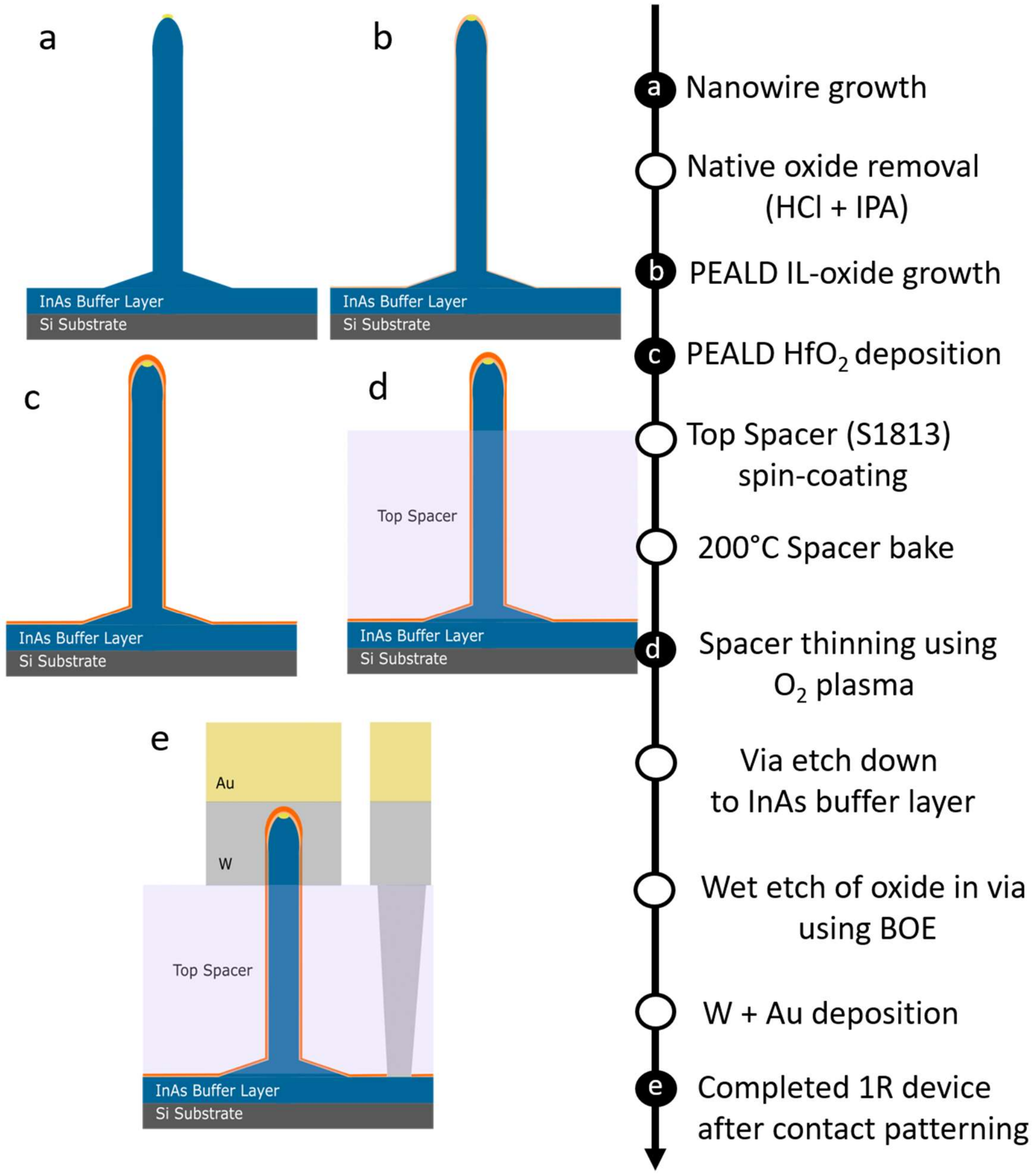

Figure S1. Fabrication flow diagram illustrating the cross-sectional structure of the InAs vertical nanowire RRAM. The key process steps include: a) InAs nanowire growth which is used as the RRAM top electrode (TE) b) IL-oxide growth by controlling the oxygen plasma pulse length (t_{plasma} = 80 s for first 10 cycles) during plasma-enhanced atomic layer deposition c) The remaining PEALD cycles are used to grow the 2.8 nm-thick HfO_2 d) RRAM area definition by controlling the top spacer (S1813) thickness. The spacer is thinned down using dry etching with an O_2 plasma e) Sputter deposition and patterning of W for RRAM bottom electrode (BE) and Au for contact pads for easier probing.

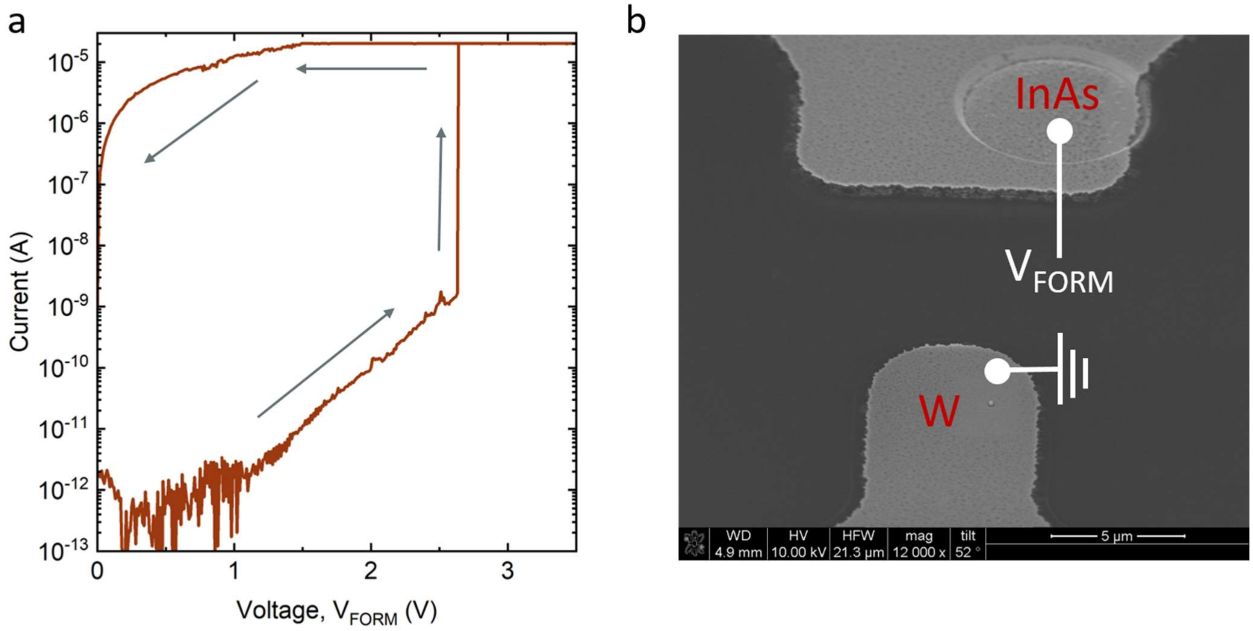

Figure S2. a) I-V characteristics for the initial filament forming with $V_{\mathrm{FORM}} \sim 3.0$ V b) A scanning electron microscope image showing the top view of the contact pads for the InAs vertical nanowire RRAM along with the biasing conditions. The InAs acts as the electrical top electrode where VFORM is applied and contacted through the via going down to InAs. The W top electrode lays on top of the resist spacer and it is forced to ground.

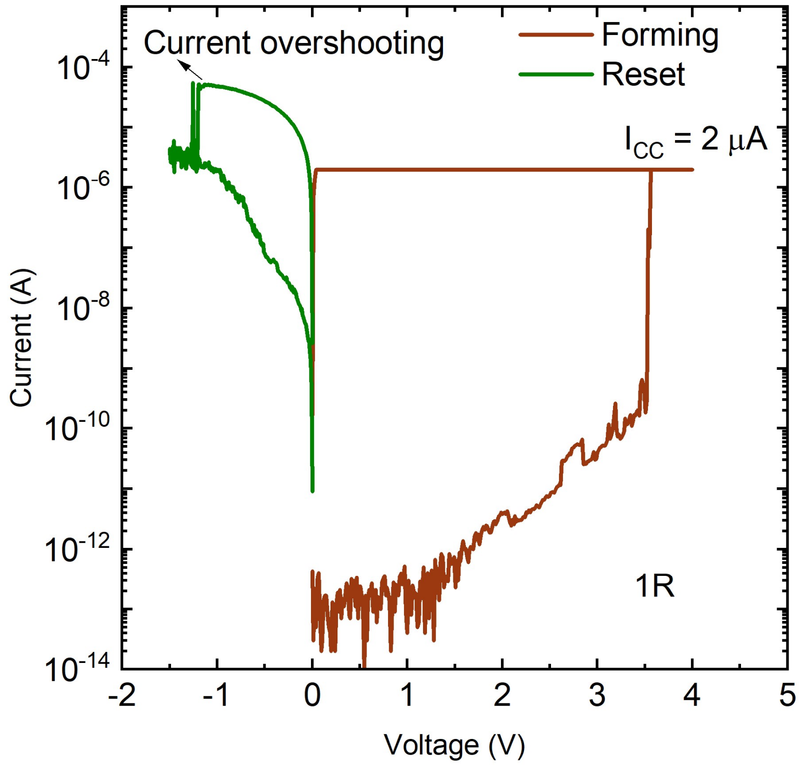

Figure S3. I-V characteristics for the initial filament forming (V_{FORM}) followed by the 1st reset operation. The forming was carried out with a compliance current of 2.0 μA in this case. It can be noted from the reset operation that the low resistance state (LRS) current is a lot larger indicating unwanted filament growth. This leads to a very abrupt reset and larger spread in HRS while cycling [1]. The current overshoot problem can be avoided by having a current limiting device such as a MOSFET selector in series with the RRAM [2].

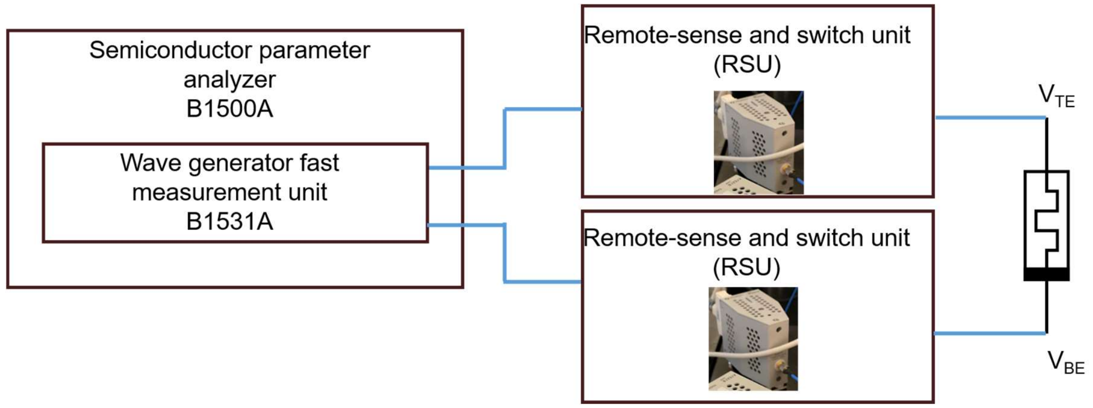

Figure S4. Measurement setup used to carry out low frequency noise (1/*f*-noise) and random telegraph noise measurements. The InAs VNW RRAM TE and BE were connected to RSU units and a fast current measurement was made. A similar setup was used in ref. 3.

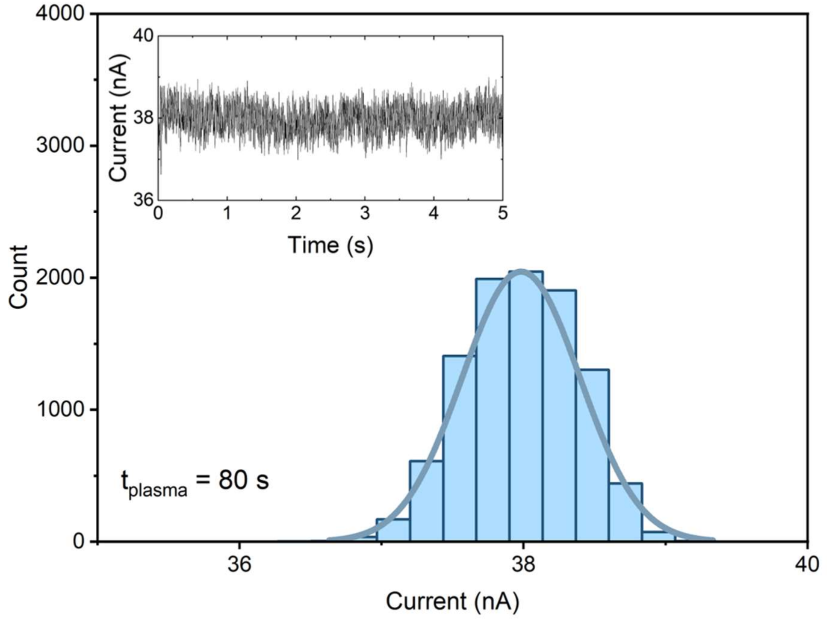

Figure S5. *Current histogram for the VNW RRAM for* t_{plasma} *= 80 s clearly indicating only one current level. The inset shows the measured current at* V_{READ} *= 500 mV*

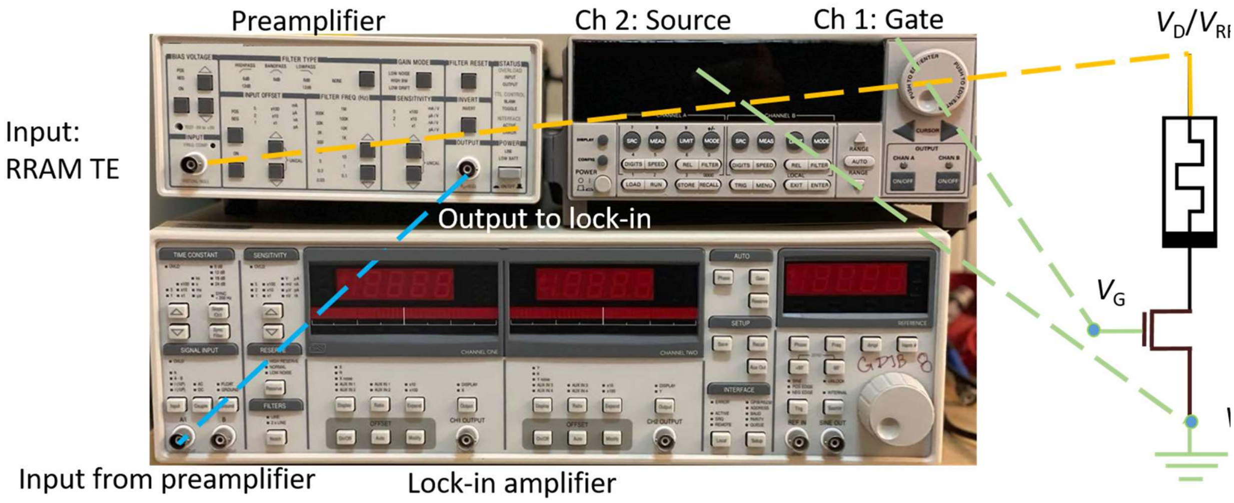

Figure S6. Measurement setup using a lock-in amplifier and a preamplifier to measure the InAs VNW 1T1R cell. The input to the RRAM TE/MOSFET drain is supplied by the preamplifier. The lock-in amplifier is used to record the voltage noise fluctuations as a function of frequency. The same setup is used in [4] [5].

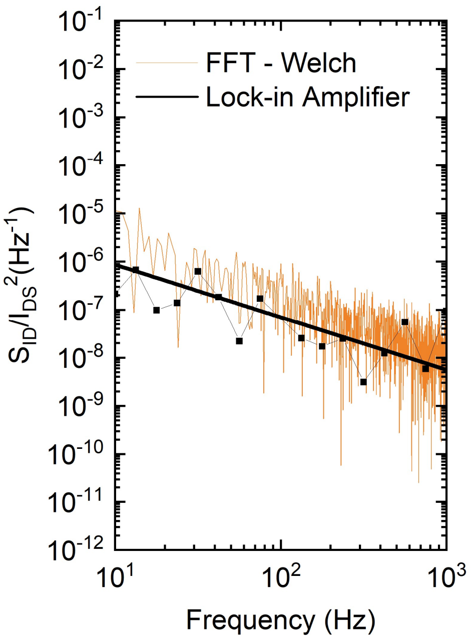

Figure S7. The current noise power spectral density measured on an InAs VNW MOSFET in a 1T configuration measured using both setups as shown in supplementary Fig. IV and Fig. V. It can be noted from the measurement that both setups give similar results.

References

[1] Kim, M.-H.; Kim, S.; Bang, S.; Kim, T.-H.; Lee, D. K.; Cho, S.; Park, B.-G. Uniformity Improvement of Sin *x* -Based Resistive Switching Memory by Suppressed Internal Overshoot Current. *IEEE Transactions on Nanotechnology* 2018, *17* (4), 824–828.

[2] Ram, M. S.; Persson, K.-M.; Irish, A.; Jönsson, A.; Timm, R.; Wernersson, L.-E. High-Density Logic-in-Memory Devices Using Vertical Indium Arsenide Nanowires on Silicon. Nature Electronics 2021, 4 (12), 914–920.

[3] Watanabe, S.; Sugawara, H.; Häusermann, R.; Blülle, B.; Yamamura, A.; Okamoto, T.; Takeya, J. Remarkably Low Flicker Noise in Solution-Processed Organic Single Crystal Transistors. Communications Physics 2018, 1 (1).

[4] Ram, M. S; J. Svensson, S. Skog, S. Johannesson and L. -E. Wernersson, "Low-frequency Noise in Vertical InAs/InGaAs Gate-all-around MOSFETs at 15 K for Cryogenic Applications," in IEEE Electron Device Letters, 2022

[5] Hellenbrand, M.; Memisevic, E.; Berg, M.; Kilpi, O.-P.; Svensson, J.; Wernersson, L.-E. Low-Frequency Noise in III–V Nanowire Tfets and Mosfets. IEEE Electron Device Letters 2017, 38 (11), 1520–1523.
